# The role of the Annexin-A1/FPR2 system in the regulation of mast cell degranulation provoked by compound 48/80 and in the inhibitory action of nedocromil

**DOI:** 10.1016/j.intimp.2016.01.003

**Published:** 2016-03

**Authors:** Ajantha Sinniah, Samia Yazid, M. Perretti, Egle Solito, R.J. Flower

**Affiliations:** a Molecular Therapy Department, Institute of Ophthalmology, 11/14 Bath Street, London EC1V 9EL, UK; b Centre for Translational Medicine and Therapeutics, William Harvey Research Institute, St Barts and the Royal London School of Medicine, Queen Mary University of London, Charterhouse Square, London EC1M 6BQ, UK; c Centre for Biochemical Pharmacology, William Harvey Research Institute, St Barts and the Royal London School of Medicine, Queen Mary University of London, Charterhouse Square, London EC1M 6BQ, UK

**Keywords:** Mast cell, Cromones, Compound 48/80, ANX-A1, PKC, PP2A

## Abstract

1. We investigated the role of Annexin (ANX)-A1 and its receptor, ALX/FPR2, in the regulation of mast cell degranulation produced by compound 48/80.
2. Both human cord-blood derived mast cells (CBDMCs) and murine bone marrow derived mast cells (BMDMCs) release phosphorylated ANX-A1 during treatment with glucocorticoids or the mast cell ‘stabilising’ drugs ketotifen and nedocromil.
3. Compound 48/80 also stimulated ANX-A1 phosphorylation and release and this was also potentiated by nedocromil. Anti-ANX-A1 neutralising monoclonal antibodies (Mabs) enhanced the release of pro-inflammatory mediators in response to compound 48/80.
4. Nedocromil and ketotifen potently inhibited the release of histamine, PGD_2_, tryptase and β-hexosaminidase from mast cells challenged with compound 48/80. Anti-ANX-A1 neutralising Mabs prevented the inhibitory effect of these drugs.
5. BMDMCs derived from Anx-A1^−/−^ mice were insensitive to the inhibitory effects of nedocromil or ketotifen but cells retained their sensitivity to the inhibitory action of hu-r-ANX-A1.
6. The fpr2/3 antagonist WRW4 blocked the action of nedocromil on PGD_2_, but not histamine, release. BMDMCs derived from fpr2/3^−/−^ mice were insensitive to the inhibitory effects of nedocromil on PGD_2_, but not histamine release.
7. Compound 48/80 stimulated both p38 and JNK phosphorylation in CBDMCs and this was inhibited by nedocromil. Inhibition of p38 phosphorylation was ANX-A1 dependent.
8. We conclude that ANX-A1 is an important regulator of mast cell reactivity to compound 48/80 exerting a negative feedback effect through a mechanism that depends at least partly on the FPR receptor.

## Highlights

• Mast cell ‘stabilisers’ release anti-inflammatory ANX-A1 from mast cells.
• Anti-ANX-A1 neutralising Mabs blocked their inhibitory effect.
• Mast cells from Anx-A1^−/−^ mice are insensitive to nedocromil or ketotifen.
• The effect of ANX-A1 on PGD_2_ release depends upon the fpr2/3 receptor.
• ANX-A1, acting partly through FPR, is a key regulator of mast cell reactivity.

## 1 Introduction

Glucocorticoids (GCs) exert many acute anti-inflammatory effects through the release of the 37 kDa protein Annexin-A1 (ANX-A1; reviewed in [1], [2]). In addition to increased transcription of Anx-A1 mRNA [3], exposure of target cells to GCs transiently activates PKC through a non-genomic glucocorticoid receptor-dependent mechanism [4] and phosphorylation of Serine-27 in the ANX-A1 N-terminal domain by activated PKC, promotes secretion of extracellular ANX-A1. FPR receptors appear to be the main extracellular target for this protein [5], [6], [7] with anti-inflammatory effects being triggered by ALX/FPR2 homo-dimerisation [8].

Mast cells are an abundant source of ANX-A1 and treatment with anti-inflammatory glucocorticoids such as dexamethasone [9] or induction of inflammation [10], [11], [12] provokes rapid Anx-A1 mRNA and/or new protein synthesis.

The degranulation of mast cells can be prevented by glucocorticoids utilising both a ‘fast’ and ‘slow’ mechanisms [13] as well as by the cromone group of drugs [14]. We have demonstrated that these latter drugs can act synergistically with glucocorticoids in U937 cells by inhibiting the PP2A phosphatase enzyme (EC 3.1.3.53) that normally limits the activation of PKC. This prolongs the time course of ANX-A1 phosphorylation, increasing its release [15] and hence exerting an increased inhibitory action.

This mechanism underlies the ability of these drugs to inhibit neutrophil migration and mitigate intestinal reperfusion damage in the mouse [14] as well as the inhibition of degranulation of human cord blood-derived mast cells (CBDMCs) provoked by IgE cross-linking [16]. Congruent with this notion, nedocromil is inactive in preventing compound 48/80 or IgE/anti-IgE degranulation of bone marrow derived mast cells obtained from mice lacking the Anx-A1 gene [16].

To exclude the possibility that this was an effect only relevant to signalling through the high-affinity IgE receptor, we here used compound 48/80 as a degranulating stimulus. This bypasses the high affinity IgE receptor mechanism and acts directly on G-proteins to produce mast cell degranulation. We are thus able to show that ANX-A1 is actually an endogenous regulator of mast cell function, regardless of the mode of stimulation, being phosphorylated and released during the degranulation event itself and acting either wholly or partially, through the FPR receptor system to regulate the limit and extent of mediator release. The ANX-A1-FPR system can also be activated in the absence of degranulation by drugs such as the glucocorticoids, the cromones and ketotifen, which have the ability to prevent degranulation, and this probably provides a clue to their mechanism of clinical action.

## 2 Materials and methods

### 2.1 Human cord-blood derived mast cells (CBDMCs)

CD34 + stem cells (Lonza, UK) used as a source and were cultured as previously described [16]. At week 8, un-stimulated CBDMCs were immuno-phenotyped as > 90% c-kit positive by FACS analysis. To further confirm the morphology, these cells were stained with toluidine blue which stains the metachromatic granules of the mast cells. The cells were maintained at a density of 1.0 × 10^6^ cells/ml for no longer than 15 weeks.

### 2.2 Murine bone marrow derived mast cells (BMDMCs)

To generate primary bone marrow-derived murine mast cells (BMDMCs), femur bones from Anx-A1^−/−^ BALB/C, fpr2/3^−/−^ C57/B6 and their respective WT control mice (4–6 weeks old, Charles River, Kent, UK) were used. The method has been described in detail in a previous publication [16]. The differentiation of BMDMCs takes 4–6 weeks and phenotypic maturity of these cells was checked before using the cells for in-vitro experiments.

All animal work was performed according to the UK Home Office regulations (Guidance on the Operation of Animals, Scientific Procedures Act 1986) and was approved by the Queen Mary University of London Ethics Committee (London, UK). Human cells were prepared according to a protocol approved by the East London & The City Local Research Ethics Committee (no. 06/Q605/40; P/00/029 ELCHA, London UK).

### 2.3 Treatment with drugs

CBDMCs/BMDMCs were stimulated with compound 48/80 (10 μg/ml; Sigma-Aldrich, Dorset, UK) for 10 min at 37 °C with 5% CO_2_ atmosphere. Drugs, antibodies or the FPR2/ALX antagonist (WRW4 peptide; EMD Chemicals, Darmstadt, Germany) tested in this system were added 5 min prior to the activation of compound 48/80. Samples were centrifuged at 1000 *g* for 5 min before the cell-free supernatants were collected to measure histamine and PGD_2_ release. Cell lysates and supernatant were prepared for Western blots. Sample aliquots were stored at − 80 °C for further analysis. Drugs such as the anti-allergic nedocromil or the glucocorticoid dexamethasone were added as required. In some cases the drug-treated cell aliquots were incubated with a well-characterized specific neutralising anti-ANX-A1 (clone 1B; 20 μg/ml), or an equivalent concentration of an isotype matched irrelevant (IgG1, ABD Serotec, Oxford, UK), mAb.

### 2.4 Measurement of histamine release

A commercially available enzyme immunoassay was used to detect and quantify histamine-released in the supernatant (SPI bio, Strasbourg, France). The assay was conducted following the manufacturer's protocols. A standard curve ranging from 0.39–50 nM histamine was prepared using the reagent provided and the optical density was then read within 60 min in a microplate reader (Titertek™, Vienna, Austria) at 405 nm. In some cases, the total cell content of histamine was established by freeze-thawing of cells prior to challenge.

‘Spontaneous histamine release’ was expressed as a percentage of the amount (nmol) of histamine released in the absence of challenge divided by the total histamine content of cells.

The ‘net histamine release’ was expressed as a percentage of the amount (nmol) histamine released during challenge (corrected for the spontaneous release) divided by the total histamine content of cells (corrected for the spontaneous release).

### 2.5 Measurement of PGD_2_ release

A commercially available enzyme immunoassay (Cayman Chemical, Michigan, USA) was used to detect and quantify PGD_2_ released in the supernatant. The assay was conducted following the manufacturer's protocols. A standard curve ranging from 78 to 10,000 pg/ml PGD_2_ was prepared using the reagent provided and the optical density was then read within 60 min in a microplate reader (Titertek™, Vienna, Austria) at 405 nm.

### 2.6 Assessment of Ser^27^ ANX-A1-P, PKC, p38, JNK and tryptase by Western blotting

Ser^27^ ANX-A1-P and PKC were assessed as previously described [16]. Total tryptase was assessed by using mouse monoclonal anti-tryptase antibody (1:1000; Abcam, UK). Expression of phospho- and total JNK and p38 MAPK (1:1000; Cell Signaling Technology, New England Biolabs UK Ltd, Hitchin, UK) was assessed by Western blot, as described previously [15]. A horseradish peroxidase-conjugated secondary antibody (1:5000; Sigma-Aldrich, Poole, UK) detected bands related to the proteins of interest and these were revealed using ECL reagents and quantitated using the Image J densitometry program. All data were normalised to total protein and expressed as percentage of control.

### 2.7 Subcellular distribution of PKC and ANX-A1 in cord blood derived mast cells (CBDMCs)

CBDMCs were plated at a density of 2 × 10^5^ cells in a 1.5 ml Eppendorf tube. Cells were treated with the stipulated drugs for 5 min prior to stimulation with compound 48/80. 2% PFA in 0.1 M PBS was added into the Eppendorf tube for 10 min on ice to fix the cells. Since the CBDMCs are non-adherent cells, each immunostaining steps were performed in 1.5 ml Eppendorf tube and centrifuged at 1000 *g* for 5 min between each methodological step and the supernatant was aspirated carefully with a vacuum without disrupting the pellet. After fixation, the cells were washed with 0.1 M PBS. Cells were then permeabilised with 0.1% Triton X-100 in PBS for 5 min. Non-specific secondary antibody binding site were blocked by incubation of 10% FCS in PBS for 30 min. Primary antibodies (ANX-A1, α-rabbit, 1:1000, Invitrogen; α-mouse, 1:100, Abcam and total PKC α-rabbit, 1:50, Cell Signaling Technology) was diluted with 1% FCS in PBS and left to incubate at 4 °C overnight. The cells were washed twice with 1% FCS in PBS and incubated with fluorescent-labelled secondary antibody (Alexa Fluor 488, green and Alexa Fluor 546, red) for 1 h at room temperature. The cells were then washed with PBS and stained with 100 ng/ml DAPI (Invitrogen) in ddH_2_O for 5 min. The cells were washed with 30 μl ddH_2_O and dropped onto the slide using a pipette. Cover slips were carefully mounted onto the slides using the Prolong Gold Mountant (Invitrogen). Slides were then analysed and micrographs were taken using the confocal microscope (Leica) at 63 × magnification.

### 2.8 Statistical analyses

Where the data satisfied the conditions (e.g. Gaussian distribution), we used ANOVA with the Bonferroni post-hoc correction. In some cases where only two columns of data were analysed, we used Student's T test (unpaired).

### 2.9 Drugs and reagents

Nedocromil sodium was generously supplied by Sanofi-Aventis (Paris, France), dexamethasone was obtained from Sigma-Aldrich (Poole, Dorset, UK). The drugs were made freshly on the day of every experiment.

## 3 Results

### 3.1 Prolongation of GC-induced PKC phosphorylation by nedocromil

We have previously observed that PKC activation is responsible for the phosphorylation of ANX-A1 in the U937 cells [15] and the same phenomenon is observed in CBDMCs [16].

GCs such as dexamethasone activate PKC by a non-genomic receptor based mechanism probably involving PI3 kinase [4], [17] and in U937 cells we have determined that nedocromil synergises with effects of dexamethasone by prolonging PKC phosphorylation secondary to inhibition of the PP2A phosphatase [15].

We confirmed our previous observation that nedocromil action is dependent upon PKC by testing the effect of an inhibitor of PKC on the effect of nedocromil on the appearance of ANX-A1 Ser-27 in CBDMCs. Using a densitometry analysis (n = 3; Suppl. Fig. 1) we found that the PKC inhibitor Gö 6983 (10 μM) not only reduced the basal level of ANX-A1 Ser-27 from 104.6 ± 2.4 AD to 29.88 AD units but also completely reversed the potentiation of phosphorylation (1.3-fold) seen after treatment with 10 nM nedocromil reducing it from 135.3 ± 1.1 units to 48.6 ± 8.5 AD units.

We chose 5 min time point to treat CBDMCs with nedocromil as we have shown previously that the optimal effect of nedocromil was seen by 5 min at which time the amounts of ANX-A1 Ser-27 had increased by 2.1 ± 0.20 fold and the amounts of phospho-PKC by 3.6 ± 0.23 fold [16].

We did not measure inhibition of PP2A by nedocromil directly in this study (although we did so in a previous publication [15]) but instead used a surrogate measure to check that this drug acts in a manner consistent with our previous reports. PP2A terminates the activity of PKC thus reducing its dwell time at the plasma membrane when activated. To ascertain whether this was seen in CBDMCs, we pre-treated these cells with either 10 nM of nedocromil, 2 nM of dexamethasone or both drugs in combination for various time points (0–60 min).

**Fig. 1:**
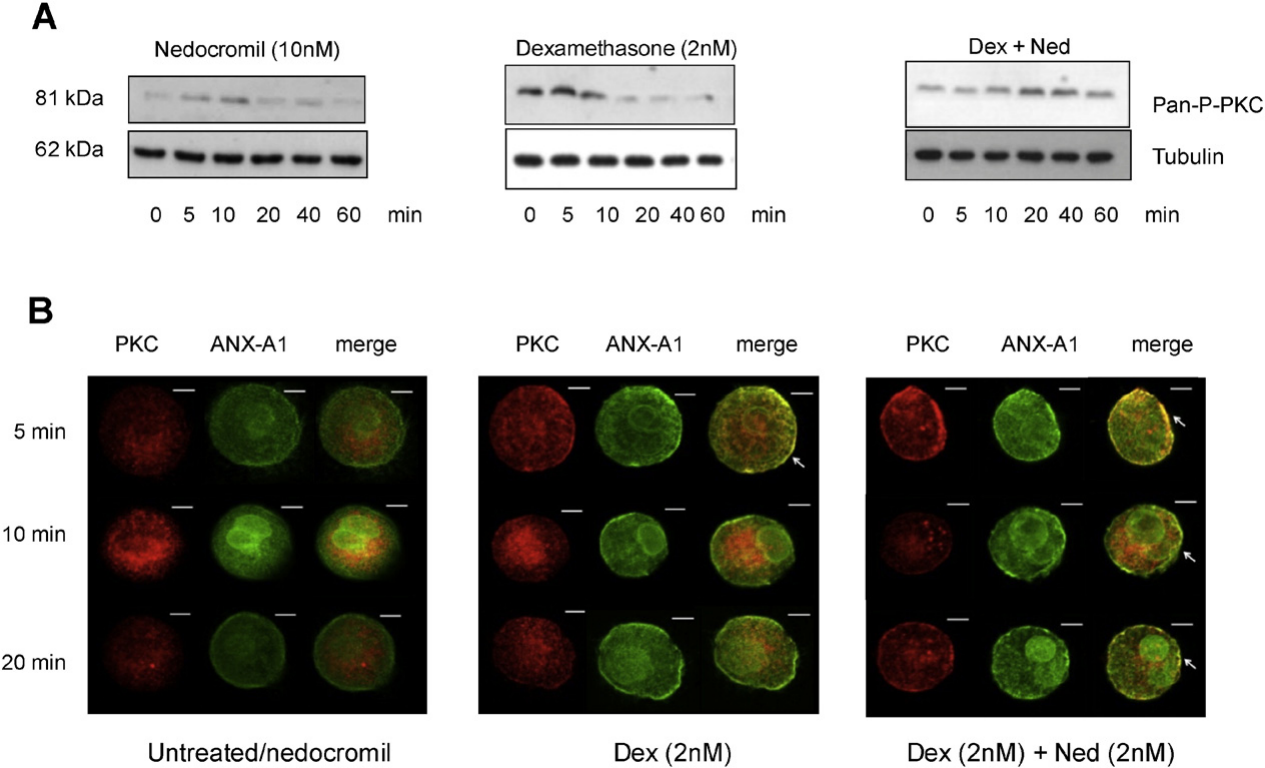
Nedocromil prolongs the dexamethasone stimulation of PKC phosphorylation and increases the dwell time of PKC at the CBDMC plasma membrane. Panel A: Western blot analysis of PKC phosphorylation at different time points in CBDMCs treated with nedocromil (10 nM; LH block), dexamethasone (2 nM; centre) or both in combination (RH block). Increased PKC phosphorylation is detectable at 5 min and peaks at 10 min when CBDMCs were treated with nedocromil and at 5 min following treatment with dexamethasone. In the presence of both dexamethasone and nedocromil, phosphorylation of PKC is potentiated by 4 fold at 40 min. Blots are a representative of 3 independent experiments. Panel B: The confocal images show a complementary experiment to that in panel A. The three columns represent the information from the two colour channels and the merged channel. Cells were stained for total PKC (red; Alexa Fluor 546; anti-rabbit) and total ANX-A1 1B (green; Alexa Fluor 488; anti-mouse). The micrographs of untreated CBDMCs (left hand block) show similar cytoplasmic distribution of total PKC and ANX-A1 across various time points. There is some localization of ANX-A1, but not PKC, at the membrane. The effect of nedocromil alone on the co-localisation of ANX-A1 and PKC was undetectable and indistinguishable from untreated cells. Cells treated with dexamethasone (middle block) for 5 min shows that there is some co-localisation of PKC and ANX-A1 at membrane (white arrow). However, this had disappeared by 10 min. Cells treated with both dexamethasone and nedocromil for 5 min (right hand block) shows that there is a striking degree of co-localisation at the membrane level between total PKC and total ANX-A1 (white arrows) and this persisted for 20 min, suggesting that nedocromil prolonged the activity of PKC at the membrane level probably by inhibiting the PP2A enzyme. The images were taken at 63 × oil magnification. Scale bars: 10 μm. This composite figure is representative of three independent experiments.

Fig. 1 (panel A), shows that, as expected, treatment with nedocromil alone provoked very weak PKC phosphorylation that was maximal by 5–10 min. Treatment with dexamethasone alone produced a burst of PKC phosphorylation which resulted in some co-localisation of ANX-A1 and PKC at the cell membrane although this was finished by 5 min. However, when both drugs were used in combination the time course of PKC phosphorylation was prolonged reaching a maximum at 20–40 min.

When activated, PKC translocates to the plasma membrane where it is co-located with its substrates (in this case ANX-A1) and where it is subsequently inactivated by PP2A prior to recycling and/or ubiquitination by the cell [18]. Any prolongation of PKC activity due to inhibition of PP2A would therefore be reflected by an increased dwell time of the enzyme in association with ANX-A1 at the cell membrane. Fig. 1 panel B shows an experiment where analysed the effects of these drugs on PKC dwell time in CBDMCs using confocal microscopy.

The CBDMCs were stained for total PKC (red; Alexa Fluor 546; anti-rabbit) and total ANX-A1 (green; Alexa Fluor 488; anti-mouse). CBDMCs were treated with dexamethasone (2 nM) alone or in combination with nedocromil (10 nM) across various time points. In this experiment, nedocromil produced barely detectable activation of PKC alone compared with untreated cells (not shown) and similar cytoplasmic distribution of total PKC and ANX-A1 was seen at 5, 10 and 20 min (left hand composite). PKC was observed mainly in the cytoplasmic or peri-nuclear region apparently associated with vesicles. Some ANX-A1 was clearly localised at the plasma membrane but there was no co-localisation with PKC. However, when CBDMCs were treated with dexamethasone for 5 min (central composite), some co-localisation at membrane level between total PKC and ANX-A1 at 5 min but this effect was not maintained. Following exposure to both nedocromil and dexamethasone (right hand composite) substantial amounts of PKC translocated to the plasma membrane and co-localised with ANX-A1 and this effect was still observed at 20 min.

### 3.2 Compound 48/80 promotes Anx-A1 phosphorylation and release in a concentration dependent manner in CBDMCs

We next tested the ability of compound 48/80 alone to release histamine from CBDMCs. Since preliminary experiments suggested that 20 μg/ml compound 48/80 already promoted maximal mast cell degranulation (60–80%) and we wanted to avoid possible pitfalls associated with maximal stimulation of cells, we investigated the effect of lower concentrations of compound 48/80 ranging from 0.01–10 μg/ml.

**Fig. 2:**
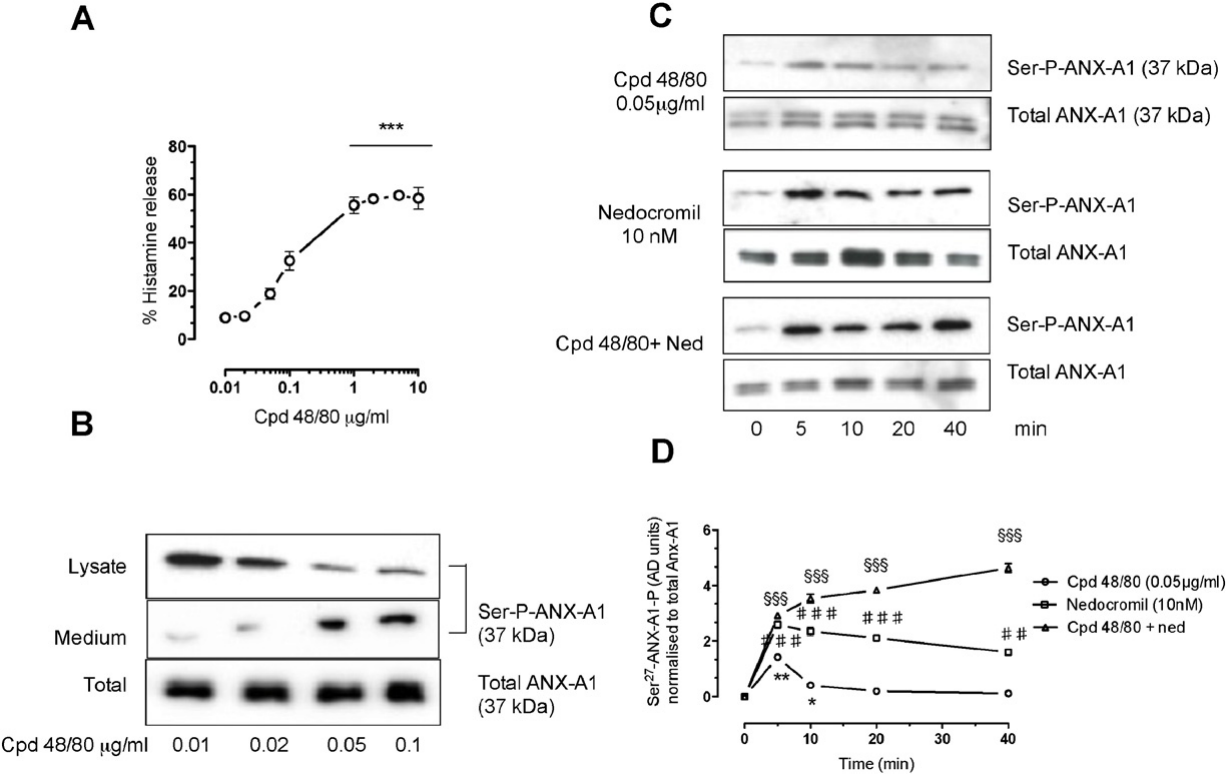
Compound 48/80 itself stimulates histamine release, PKC and Anx-A1 phosphorylation in CBDMCs in a concentration-dependent manner and this is potentiated by nedocromil. Panel A. Exposure of CBDMCs to compound 48/80 (0.01–0.1 μg/ml) caused a concentration-dependent release of histamine reaching a maximum (~ 55% total cellular histamine) at a concentration of 10 μg/ml. Data are expressed as mean ± SEM of n = 3 experiment (***p < 0.001). Panel B. Exposure of CBDMCs to compound 48/80 (0.01–0.1 μg/ml) is accompanied by a concentration-dependent increase in Ser-27 phospho ANX-A1 and release of the protein from the cell into the surrounding medium (representative example from 3 blots). Panel C. When assessed by Western blotting techniques, the phosphorylation of ANX-A1 induced by nedocromil (10 nM) or compound 48/80 (0.05 μg/ml) alone decreases after 10 min, however when the two are combined, the effect of compound 48/80 persists for 30 min (representative example from 3 blots). Panel D. A graphical representation of the densitometry analysis of panel C illustrating this effect. The values are expressed as mean ± SEM (n = 3). *p < 0.05 and **p < 0.01 vs untreated samples; ^♯♯^ p < 0.01 and ^♯♯♯^ p < 0.001 vs compound 48/80 values; ^§§§^p < 0.001 vs compound 48/80 + nedocromil values).

Fig. 2 panel A, shows that compound 48/80 produces a linear increase in histamine release from concentrations ranging from 0.01 to 0.1 μg/ml, but that thereafter the effect reaches a plateau at ~ 60% of net histamine release in concentrations from 1 to 10 μg/ml. As concentrations of compound 48/80 in the range of 0.01–0.1 μg/ml promote only about 10–30% of net histamine release, we used these concentrations to investigate the ability of compound 48/80 to induce Anx-A1 phosphorylation.

Fig. 2 panel B, shows the disposition of intra- and extracellular phospho ANX-A1 in response to graded concentrations of compound 48/80 as assessed by Western blotting. Compound 48/80 induces a concentration-dependent increase in ANX-A1 phosphorylation at the Ser-27 residue, and promotes its release into the medium even in concentrations when it produces only small effects (< 20%) on histamine release.

The time course of the response to compound 48/80 (0.05 μg/ml) was tested in conjunction with nedocromil in Fig. 2 panels C and D. Here, Western blotting quantitated by densitometry assessed the total Ser-27 phospho ANX-A1. The effect of compound 48/80 (1.4 fold increase) was observed to be transient with a maximum at 5 min. The effect of 10 nM nedocromil (2.7 fold increase) was also maximal at 5 min although it was still detectable at 40 min. However, a combination of both agents produced an intense (4.5 fold increase) and a long lasting (> 40 min) stimulation of ANX-A1 phosphorylation.

From these experiments we conclude that 48/80, in addition to activating CBDMC degranulation, also stimulates ANX-A1 phosphorylation and that this is itself prolonged by the action of nedocromil.

### 3.3 The inhibition by nedocromil (10 nM) of compound 48/80 induced histamine, PGD_2_, β-hexosaminidase and tryptase release by CBDMCs is reversed by anti-ANX-A1 neutralising antibodies

To determine the role of ANX-A1 in promoting the stability of mast cells, the effect of a specific neutralising monoclonal anti-ANX-A1 mAb was tested. We have previously reported [16] that the inhibitory effect of nedocromil on IgE/anti-IgE cross-linking induced degranulation of CBDMCs is reversed in the presence of anti-ANX-A1 mAbs. To test whether this was a general effect of degranulating agents, we here used as the stimulus compound 48/80, which bypasses the high-affinity IgE receptor dependent mechanism of cell activation.

CBDMCs were pre-incubated for 20 min with 20 μg/ml neutralising anti-ANX-A1 mAb 1B, or an equivalent concentration of an irrelevant isotype-matched mAb prior to treatment with nedocromil and compound 48/80. We also tested ketotifen as we have previously reported that this drug, which is chemically distinct from the cromones, exerts similar effects to nedocromil.

**Fig. 3:**
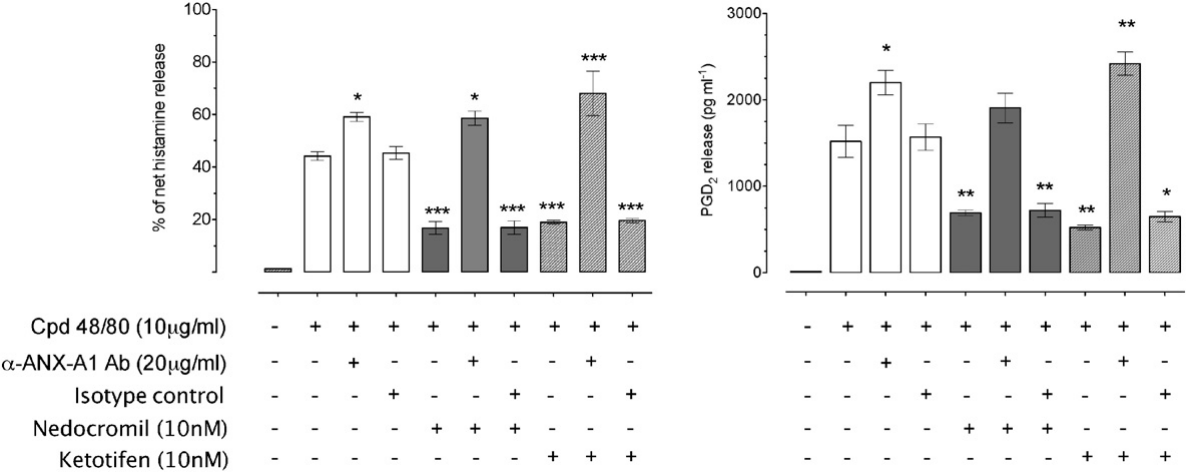
The inhibition by nedocromil and ketotifen of mediator release from CBDMCs stimulated with compound 48/80 is dependent upon Anx-A1. CBDMCs were plated at a density of 2 × 10^5^ cells per well and the stipulated groups were treated with 20 μg/ml ANX-A1 neutralising antibody or an irrelevant isotype control. Subsequently, the cells were pre-treated with nedocromil (10 nM) for 5 min followed by compound 48/80 (10 μg/ml) stimulation for 10 min. To assess the effects of ANX-A1 removal, the cells were incubated with the ANX-A1 neutralising antibody (or an irrelevant isotype control) only. The supernatants were collected from the samples and assessed for histamine (top panel) and PGD_2_ (lower panel). Nedocromil produced consistent inhibition of histamine and PGD_2_ release, but this was abrogated in the presence of the immuno-neutralising mAb, but not the control reagent. Data are expressed as mean ± SEM from n = 3 experiment and were analysed using one-way analysis of variance (ANOVA), followed by a Bonferroni post-hoc test, *p < 0.05, **p < 0.01, ***p < 0.001 vs unstimulated).

We observed (Fig. 3) that 10 min stimulation of CBDMCs with 10 μg/ml of compound 48/80 provoked the release of approximately 44% of net histamine release, and 1520 pg/ml of PGD_2_. We also measured the net β-hexosaminidase (Suppl. Fig. 2) and tryptase content of the cells (Suppl. Fig. 3) following treatment. β-Hexosaminidase release was strongly stimulated (57.4 ± 1.6% pNA units) whilst the cell content of tryptase was reduced to 65.5 ± 8.2% of control by compound 48/80 (p < 0.01).

Pre-treatment with nedocromil prior to compound 48/80 stimulation significantly (p < 0.001) inhibited the release of histamine by 73.5% and PGD_2_ by 59% (p < 0.01). Nedocromil also inhibited β-hexosaminidase release by 44% (to 32.08 ± 0.78 pNA units; p < 0.01) and reduced the release of tryptase from 65.5% to 10.1% (90.8 ± 0.90 units). However, in the presence of the neutralising anti-ANX-A1 monoclonal antibody, these inhibitory actions of nedocromil were completely abolished.

Interestingly, examination of the data suggested that the neutralising (but not the isotype-matched control) monoclonal anti-ANX-A1 mAb alone exhibited a tendency to enhance the release of mediators in the presence of compound 48/80. For example, in Fig. 3, the release of histamine stimulated by compound 48/80 was increased from 45.6 ± 2.26 to 54.52 ± 1.18% (p < 0.01) and the release of PGD_2_ from 1432.77 ± 175.77 to 1515.91 ± 127.84 pg/ml (p < 0.01).

The amount of intracellular tryptase remaining after compound 48/80 was decreased from 65.5 ± 6.83% to 53.6 ± 6.73 (although this did not achieve statistical significance) in the presence of the neutralising anti-ANX-A1 monoclonal (but not the isotype-matched control).

The release of β-hexosaminidase was also increased in the presence of the neutralising anti-ANX-A1 monoclonal from 57.37 ± 1.60 to 67.96 ± 4.44 pNA units (p < 0.01). In this connection, we also observed (Suppl. Fig. 2; panel B) that even the addition of the neutralising anti-ANX-A1 mAb to resting, unstimulated CBDMCs increased the spontaneous release of β-hexosaminidase from 8.9 ± 0.3% to 43.6 ± 3.6% (p < 0.001). All these data suggest that without some external ANX-A1, CBDMCs can undergo partial spontaneous degranulation even in the absence of an added stimulus.

### 3.4 Effect of nedocromil and ketotifen on compound 48/80 induced mediator release in BMDMCs from Anx-A1 null mice

**Fig. 4:**
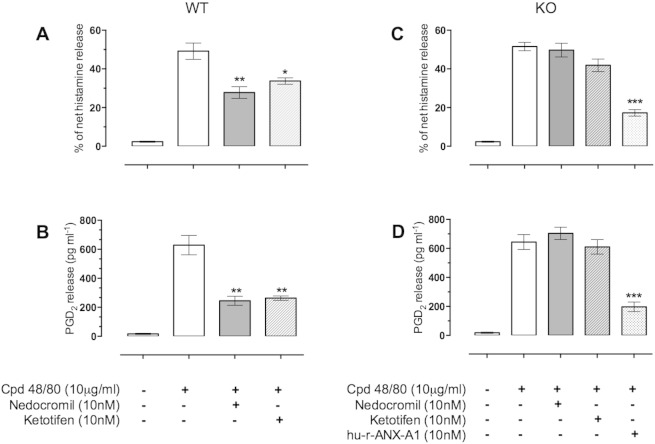
The inhibitory action of nedocromil and ketotifen is dependent upon the presence of mast cell Anx-A1. BMDMCs from both Anx-A1^+/+^ (panels A and B) and Anx-A1^−/−^ (panels C and D) mice were cultured and prepared as described. The cells were incubated with either nedocromil (10 nM) or ketotifen (10 nM) and, in the case of the Anx-A1^−/−^ cells, 10 nM hu-r-Anx-A1. From panels A and B it can be seen that both nedocromil and ketotifen inhibit histamine (43.6 and 30% respectively) as well as PGD_2_ (61.04% and 58.1% respectively). These drugs do not produce significant inhibitory actions in the Anx-A1^−/−^ cells (panels C and D) although hu-r-Anx-A1 retains its potent activity demonstrating that this is not caused by a failure of the cells to respond. *0.05 < p; **0.01; ***0.001 vs stimulated release; n = 5.

To further confirm the role of Anx-A1 in the inhibition by nedocromil and ketotifen we tested the activity of these drugs on BMDMCs derived from Anx-A1 null mice. Fig. 4 shows that both nedocromil and ketotifen are able to produce inhibition of both histamine (43.6% and 30% respectively) and PGD_2_ (61.04% and 58.1% respectively) release in wild type BMDMCs but not in cells derived from Anx-A1 null mice. However, inhibition of mediator release could still be achieved by reconstituting the system with hu-r-Anx-A1 itself, which inhibited histamine and PGD_2_ release by 66.5% and 69.34% respectively.

### 3.5 The role of the FPR receptor system in the inhibitory action of nedocromil

In many systems, the response of the cell to ANX-A1 is dependent upon the presence of the FPR receptor system (e.g. [2], [5]) and these receptors are present on mast cells (see e.g. [19], [20]). Here, we tested whether this is also true for the inhibition produced by nedocromil on histamine and PGD_2_ release.

**Fig. 5:**
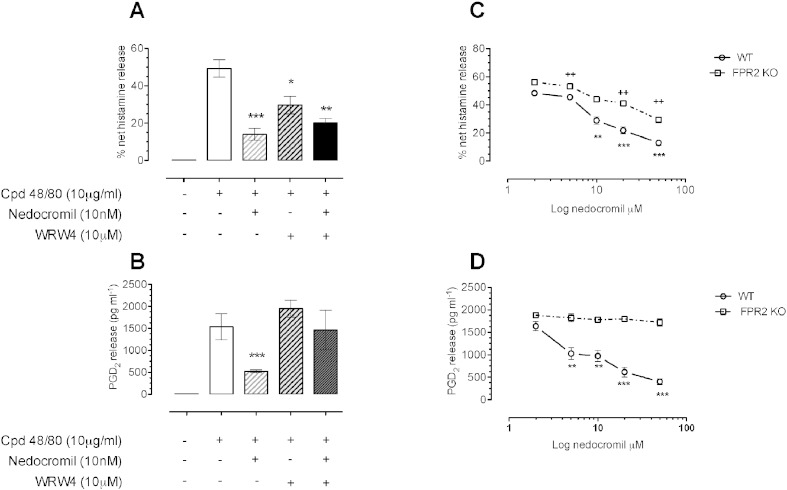
The role of FPR receptors in the inhibitory action of nedocromil in CBDMCs and BMDMCs. Nedocromil (10 nM; panel A) inhibited the release of histamine from CBDMCs stimulated with compound 48/80 (10 μg/ml). The FPR2 antagonist WRW4 (10 μM) alone partially inhibited the release of histamine but had little effect on the inhibitory action of nedocromil. In the case of PGD_2_ however (panel B), WRW4 substantially reversed the inhibitory effect of nedocromil while having no significant effect itself on the release of PGD_2_ (*0.05 < p; **0.01; ***0.001 vs stimulated release; n = 6). In the case of BMDMCs, nedocromil was found to exert a concentration-dependent inhibition of histamine release in both WT and fpr2/3 null mice (panel C) whereas nedocromil was without effect on the release of PGD_2_ from cells derived from the fpr2/3 null mice whilst producing the expected inhibition in WT cells (panel D). Data are expressed as mean ± SEM from n = 3 experiment and were analysed using one-way analysis of variance (ANOVA), followed by a Bonferroni post-hoc test, *p < 0.05, **^/^^++^ p < 0.01, ***p < 0.001 vs control.

Fig. 5, panels A and B show a set of experiments using CBDMCs in which the FPR2 antagonist WRW4 (10 μM) was used as a probe for receptor involvement. Nedocromil alone (10 nM) produced ~ 60–70% inhibition of histamine and PGD_2_ release stimulated by compound 48/80, but while the effect on PGD_2_ release was greatly reduced in the cells treated with WRW4, the inhibition of histamine release by nedocromil was not altered.

Panels C and D show experiments in which BMDMCs were prepared from fpr2/3 (the murine analogue of FPR2) null mice or WT controls and stimulated with compound 48/80. Here, a similar pattern was observed. Whilst the effect of nedocromil on histamine release was still present in the fpr2/3 null cells, it no longer produced a concentration dependent inhibition of PGD_2_ release.

### 3.6 Role of downstream signalling in nedocromil action on CBDMCs

Both JNK and p38 have been identified as important components of signalling cascades in leading to mediator release in human blood basophils [21] as well as human [22] and rat [23] intestinal mast cells, and rat basophilic leukaemia mast cells [24].

We have previously reported that activation of FPR2 homo-dimerisation in transfected HEK293 cells activated p38 MAPK pathway whereas activation of FPR1/FPR2 heterodimers activated a JNK mediated signalling pathway [8].

To test whether these signalling factors were important in the inhibitory effect of nedocromil on mast cells and whether this was Anx-A1 dependent we measured the phosphorylation of p38 and JNK in CBDMCs following compound 48/80 treatment using Western blotting techniques.

**Fig. 6:**
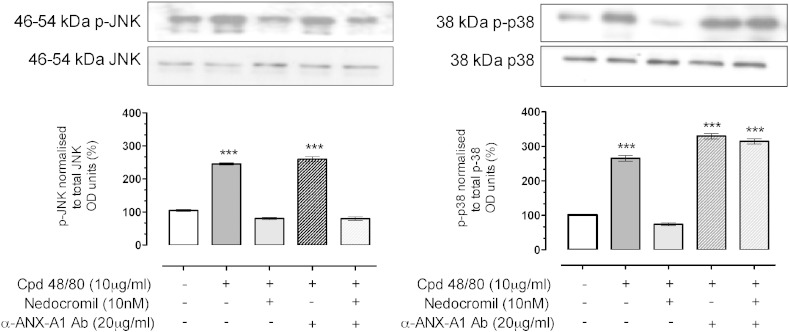
Downstream signalling in CBDMCs is differentially altered by nedocromil. Compound 48/80 (10 μg/ml) stimulation of CBDMCs increased p-38 and JNK phosphorylation, and this was inhibited by nedocromil (10 nM). The presence of ANX-A1 neutralising antibody reversed the phospho-p38 inhibitory actions of nedocromil but did not alter its inhibitory actions on JNK phosphorylation. Data are expressed as mean ± SEM (n = 3 independent experiments; ***p < 0.001 vs unstimulated).

Fig. 6 shows the results. Treatment of CBDMCs with compound 48/80 activated JNK as measured by the appearance of increased amounts of p-JNK following exposure. This was blocked if the cells were pre-treated with nedocromil and this did not appear to be ANX-A1 dependent as there was no change following incubation of the cells with the neutralising anti-ANX-A1 monoclonal. In contrast, whilst nedocromil also inhibited the activation of p38 following compound 48/80, this inhibitory activity was reversed in the presence of the anti-ANX-A1 monoclonal suggesting that activation of this signalling pathway was ANX-A1 dependent.

## 4 Discussion

Our previous publications in this area highlighted a novel mechanism of action for the cromone anti-allergic drugs. Using a U937 cell model we demonstrated [15] that the export of Anx-A1 stimulated by the glucocorticoid dexamethasone was greatly potentiated by nedocromil or cromoglycate and that this increased the biological activity of the glucocorticoid as an inhibitor of eicosanoid production by these cells. We observed that the reason for the effect was that in the presence of cromones, the stimulatory action of the liganded GR was greatly prolonged and that this appeared to be due to an inhibitory effect of these drugs on PP2A, the enzyme that normally de-activates PKC thus terminating its activity.

In a subsequent study, we demonstrated that the ability of cromones to inhibit CBDMC degranulation stimulated by IgE–anti-IgE cross-linking was prevented by immuno-neutralisation of Anx-A1 and was wholly absent in the BMDMCs from Anx-A1 null mice [16].

Whilst the cromones are known mainly as anti-allergic drugs, they have many other pharmacological actions including inhibition of leukocyte migration. We further showed [14] that the ability of cromones to produce this anti-migratory effect was not only dependent upon Anx-A1 externalisation, but also on the presence of FPR receptors on the leukocyte surface. In this study we have confirmed and extended these findings.

It has been known for many years that both PKC and PP2A are translocated to the membrane during mast cell activation whether this is caused by antigen cross-linking at the high affinity IgE receptor or agents that bypass this receptor [25], [26]. It is the phosphorylation of target proteins (such as myosin) on the cell membrane by PKC that initiates the process of secretion. PP2A, which regulates PKC activity in this respect, has been identified as a key enzyme in the regulation of mast cell reactivity and treatment of mast cells with okadaic acid inhibits secretion of mediators [26].

In this study PP2A translocation and activity was assessed indirectly in mast cells by monitoring the length of time that PKC is resident at the membrane following cell activation. Our idea that nedocromil prolongs PKC activation by decreasing the activity of PP2A enzyme was further supported by our observation here that this drug prolongs the translocation of PKC to the cell membrane. This is also consistent with previous reports in the literature that treatment of mast cells with cromones increases the phosphorylation of proteins thought to be involved in the exocytotic mechanism [27], [28].

Here we have observed that in addition to glucocorticoid/cromone treatment, compound 48/80 itself activates PKC, promotes the phosphorylation and release of ANX-A1 and that this too, is potentiated by nedocromil.

This finding points to a way by which the intensity of the degranulating stimulus is matched by an increased release of inhibitory ANX-A1 so as to regulate the overall magnitude of the secretory response. It also raises an interesting question: could nedocromil (or other cromone) actually potentiate mast cell degranulation provoked by agents such as compound 48/80 in the absence of ANX-A1?

Some support for this idea arises from our findings using immuno-neutralisation of ANX-A1. This biologic not only abrogates the inhibitory effect of both nedocromil and ketotifen but also, interestingly, we observed that there was a strong trend for the neutralising mAb alone to promote mediator secretion when the mast cells were stimulated. This certainly would fit with the general conclusion that this is an endogenous regulatory molecule.

Whilst we have previously published on the importance of ANX-A1 in the inhibitory action of cromones in mast cells stimulated by IgE–anti IgE or DNP-BSA/anti-DNP-IgE cross-linking [16], we could not rule out that this was an effect specific to signalling through the high affinity IgE receptor. In this study, we can now confirm that similar results are seen when the stimulating agent is compound 48/80, which acts directly on G proteins to stimulate the secretory event [29]. We also report here other novel findings, including the observation that inhibition of the release of other mast cell mediators such as β-hexosaminidase and tryptase by nedocromil is similarly ANX-A1 sensitive.

Of interest therefore is the notion that under conditions where ANX-A1 becomes ‘depleted’ – for example, after repetitive challenge with degranulating agents, glucocorticoids or cromones – then the inhibitory response of the latter agents will be temporarily lost. We have indeed observed this in the case of prolonged hydrocortisone stimulation of macrophages [30].

The results of the experiments in which we tested the ability of nedocromil and ketotifen to inhibit histamine and PGD_2_ release in BMDMCs is entirely congruent with the neutralising monoclonal experiments and the fact that the ANX-A1 null cells still respond to Anx-A1 establishes that the receptor system is still present on these cells. In fact, the role of the FPR receptor family in the response of mast cells to nedocromil is of particular interest. In many systems, ALX/FPR2 is the chief subtype through which ANX-A1, and other anti-inflammatory regulators such as Resolvin D_1_ and Lipoxin A_4_, exert their inhibitory actions [2], [5], [6].

Since either deletion of Anx-A1 altogether (as in the KO mouse) or its removal using neutralising monoclonals clearly establish that this protein is a vital component of nedocromil action, one would anticipate that removal of the principal receptor for Anx-A1, FPR2 (or its murine homologue) fpr2/3 would prevent the action of this drug as well.

Our observations here suggest that this is only partially true. Whilst the blockade of the FPR2 receptor in CBDMCs or its removal in the fpr2/3-null BMDMCs clearly prevented the action of nedocromil on PGD_2_ generation and release, it failed to completely block inhibitory action on histamine release.

This apparent paradox might be understood in terms of the possible involvement of other members of the FPR family. In a previous study, we observed that the inhibitory effect of nedocromil on the migration of PMN in a cell chamber model of migration was dependent upon the presence of both FPR1 and FPR2 receptors [14]. Cooray et al. [8] showed that whilst full-length ANX-A1 produced FPR2 homo-dimerisation and activated the p38 signalling pathway in HEK293 cells, *N*-Ac-2-26, an N-terminal peptide of ANX-A1, produced hetero-dimerisation of FPR1/2, and activated a different, JNK-dependent, signalling pathway.

It is therefore possible that the effect of ANX-A1 on mast cells is the result of a combination of signalling produced by the full-length protein as well as a clipped fragment such as *N*-Ac-2-26, which acts as a ligand at both FPR1 and FPR2/ALX [8]. In fact several groups have highlighted the role of proteases in the cleavage of ANX-A1 in mast cells in lung extracts from ovalbumin sensitised mice, RBL-2H3 cells [31] as well as endometrial tissue [32] so this is a distinct possibility.

The signalling data that we present also poses further questions. Many authors have commented on the roles of MAPK pathways in mast cell activation (e.g. [13]. The p38 MAPK pathway has been implicated in the generation of PGD_2_ in RBL-2H3 cells [24], sequential activation of p38 and JNK pathways is responsible for substance P induced TNF and histamine release from rat peritoneal mast cells [23]. Others have pointed out that there are apparently differences in the utilisation of MAPK members in different cells bearing the IgE receptor [21].

Given that any signalling through an ANX-A1 pathway in our system is likely through FPR receptors, our data would suggest that the effect of nedocromil on p38 activation is a direct result of signalling through the FPR receptor system, as we have observed previously [8] but that the activation of JNK is a further action of nedocromil unrelated to this signalling pathway. It is possible that this is an indirect action of nedocromil, which perhaps produces this effect by inhibition of a phosphatase at some other point in the signalling cascade.

In summary, we have highlighted evidence supporting the case that ANX-A1 is an important endogenous regulator of mast cell function, which tonically inhibits activation in a ‘resting’ state and which is secreted in increased amounts in parallel with mediators when these cells are activated and acts to limit the extent of the degranulation and activation response.

The ‘fast’ glucocorticoid response [13] in mast cells is probably brought about by the release of ANX-A1 and in the cromone anti-allergics, investigators empirically discovered a group of compounds that could exploit this mechanism by releasing ANX-A1 in the absence of degranulation, thereby inhibiting subsequent responses.

The following are the supplementary data related to this article.

Suppl. Fig. 1 Nedocromil increases in Ser27-Anx-A1 phosphorylation in CBDMCs by activating PKC. Pre-treatment of CBDMCs for 30 min with the PKC inhibitor (Gö 6983; 10 μM) reduced the basal phosphorylation of Anx-A1 as well as that induced by nedocromil (10 nM). Above: Representative blot showing the stimulation of phosphorylation by nedocromil and inhibition by the PKC inhibitor. Below: Densitometry data collected from 3 independent experiments expressed as mean ± SEM; *p < 0.05 and **p < 0.01 relative to control or samples treated with nedocromil alone.

Suppl. Fig. 2 The inhibition by nedocromil on β-hexosaminidase release from CBDMCs stimulated with compound 48/80 is dependent upon Anx-A1. In panels A, CBMCs were plated at a density of 2 × 10^5^ cells per well and the stipulated groups were treated with 10 μg/ml Anx-A1 neutralising antibody or an irrelevant isotype control. Subsequently, the cells were pre-treated with nedocromil (10 nM) for 5 min followed by compound 48/80 (10 μg/ml) stimulation for 10 min. Nedocromil produced consistent inhibition of β-hexosaminidase release, but this was abrogated in the presence of the immuno-neutralising mAb, but not the control reagent. In panel B, the cells were incubated with the Anx-A1 neutralising antibody (or an irrelevant isotype control) only. The supernatants were collected from the samples and assessed for β-hexosaminidase by colorimetric assay. Interestingly, the addition of the Anx-A1 neutralising antibody alone (but not the irrelevant isotype control) was sufficient to release β-hexosaminidase from the CMDMCs. Data are expressed as mean ± SEM from n = 3 experiment and were analysed using one-way analysis of variance (ANOVA), followed by a Bonferroni post-hoc test, *p < 0.05, **p < 0.01, ***p < 0.001 vs unstimulated. In panel A, ^§§^p < 0.01 relative to compound 48/80 treatment alone as determined by Student's T test.

Suppl. Fig. 3 The inhibitory effect of nedocromil on compound 48/80 stimulated tryptase release from CBDMCs is Anx-A1 dependent. Panel A. Confocal examination of tryptase release in CBDMCs. The cells were stained with antibodies to tryptase (green). DAPI was used to stain the nucleus (blue) and the cells were exposed to 10 μg/ml compound 48/80, 10nM nedocromil and 20 μg/ml of anti-Anx-A1 neutralising mAb. Stimulation with compound 48/80 led to distinct degranulation of the mast cells. Tryptase was confined in the cytoplasm when CBMCs were treated with nedocromil but in the presence of Anx-A1 neutralising antibody, more tryptase were released from the cell. These images are representative of 3 independent experiments. Confocal images were taken at × 63 oil magnification. Scale bars: 10 μm. Panel B. The densitometry analysis of the western blot shows that in the presence of compound 48/80, the tryptase level in the cell lysate is significantly reduced (p < 0.05). When the CBMCs were pre-treated with nedocromil (10 nM), the tryptase is retained in the cells. In the presence of Anx-A1 neutralising antibody alone or combined with nedocromil, the tryptase release is significantly (p < 0.05) enhanced. Data are expressed as mean ± SEM (n = 3, **p < 0.01, ***p < 0.001 vs unstimulated).

## Conflicts of interest

The authors declare none.
