# Supplementary figures and images for: The role of the Annexin-A1/FPR2 system in the regulation of mast cell degranulation provoked by compound 48/80 and in the inhibitory action of nedocromil
Source: Int Immunopharmacol. 2016 Mar;32:87–95. doi: 10.1016/j.intimp.2016.01.003 (PMC4760273; doi:10.1016/j.intimp.2016.01.003)

# Suppl Figure 1

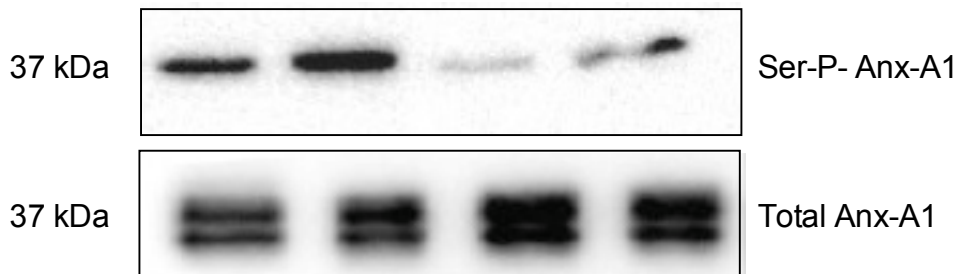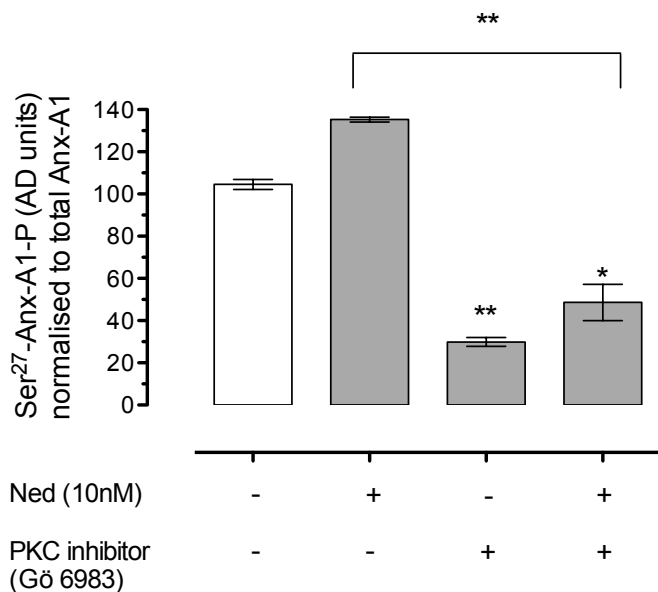

Supplement: Suppl. Fig. 1 — Nedocromil increases in Ser27-Anx-A1 phosphorylation in CBDMCs by activating PKC. Pre-treatment of CBDMCs for 30 min with the PKC inhibitor (Gö 6983; 10 μM) reduced the basal phosphorylation of Anx-A1 as well as that induced by nedocromil (10 nM). Above: Representative blot showing the stimulation of phosphorylation by nedocromil and inhibition by the PKC inhibitor. Below: Densitometry data collected from 3 independent experiments expressed as mean ± SEM; *p < 0.05 and **p < 0.01 relative to control or samples treated with nedocromil alone. [file mmc1.pdf]

# Suppl. Figure 2.

**A.**

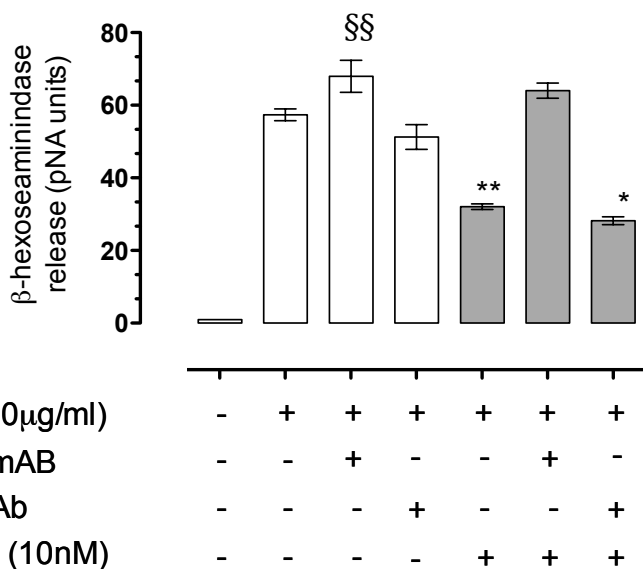

**B.**

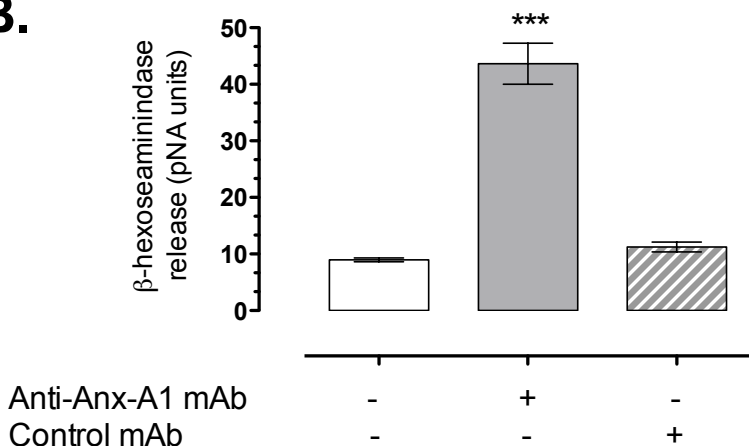

Supplement: Suppl. Fig. 2 — The inhibition by nedocromil on β-hexosaminidase release from CBDMCs stimulated with compound 48/80 is dependent upon Anx-A1. In panels A, CBMCs were plated at a density of 2 × 10^5^ cells per well and the stipulated groups were treated with 10 μg/ml Anx-A1 neutralising antibody or an irrelevant isotype control. Subsequently, the cells were pre-treated with nedocromil (10 nM) for 5 min followed by compound 48/80 (10 μg/ml) stimulation for 10 min. Nedocromil produced consistent inhibition of β-hexosaminidase release, but this was abrogated in the presence of the immuno-neutralising mAb, but not the control reagent. In panel B, the cells were incubated with the Anx-A1 neutralising antibody (or an irrelevant isotype control) only. The supernatants were collected from the samples and assessed for β-hexosaminidase by colorimetric assay. Interestingly, the addition of the Anx-A1 neutralising antibody alone (but not the irrelevant isotype control) was sufficient to release β-hexosaminidase from the CMDMCs. Data are expressed as mean ± SEM from n = 3 experiment and were analysed using one-way analysis of variance (ANOVA), followed by a Bonferroni post-hoc test, *p < 0.05, **p < 0.01, ***p < 0.001 vs unstimulated. In panel A, §§p < 0.01 relative to compound 48/80 treatment alone as determined by Student's T test. [file mmc2.pdf]

# Suppl. Figure 3.

**A.**

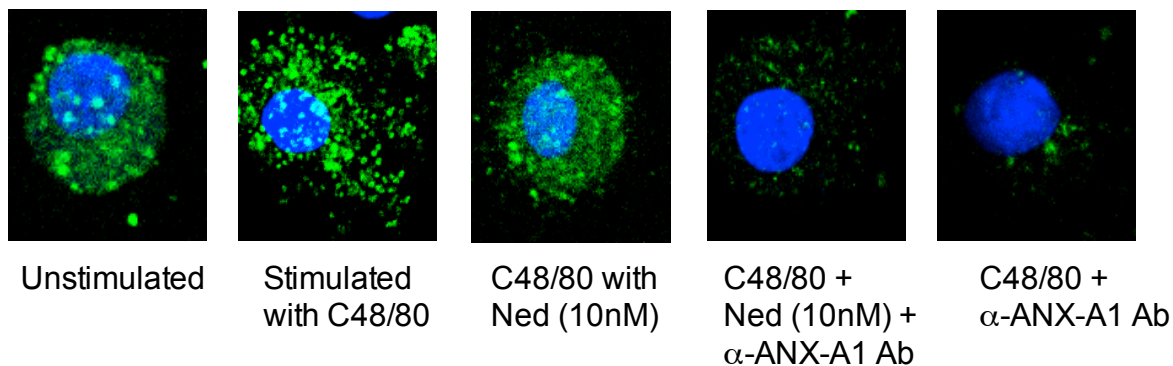

**B.**

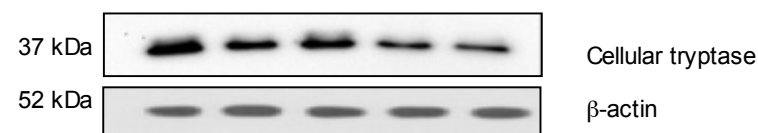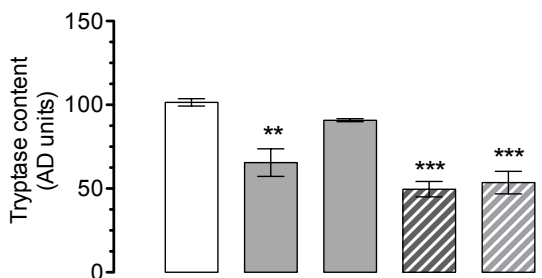

|                                 |   |   |   |   |   |
|---------------------------------|---|---|---|---|---|
| Cpd 48/80 (10 $\mu$ g/ml)       | - | + | + | + | + |
| Anti-Anx-A1 mAb (20 $\mu$ g/ml) | - | - | - | + | + |
| Nedocromil (10nM)               | - | - | + | + | - |

Supplement: Suppl. Fig. 3 — The inhibitory effect of nedocromil on compound 48/80 stimulated tryptase release from CBDMCs is Anx-A1 dependent. Panel A. Confocal examination of tryptase release in CBDMCs. The cells were stained with antibodies to tryptase (green). DAPI was used to stain the nucleus (blue) and the cells were exposed to 10 μg/ml compound 48/80, 10nM nedocromil and 20 μg/ml of anti-Anx-A1 neutralising mAb. Stimulation with compound 48/80 led to distinct degranulation of the mast cells. Tryptase was confined in the cytoplasm when CBMCs were treated with nedocromil but in the presence of Anx-A1 neutralising antibody, more tryptase were released from the cell. These images are representative of 3 independent experiments. Confocal images were taken at × 63 oil magnification. Scale bars: 10 μm. Panel B. The densitometry analysis of the western blot shows that in the presence of compound 48/80, the tryptase level in the cell lysate is significantly reduced (p < 0.05). When the CBMCs were pre-treated with nedocromil (10 nM), the tryptase is retained in the cells. In the presence of Anx-A1 neutralising antibody alone or combined with nedocromil, the tryptase release is significantly (p < 0.05) enhanced. Data are expressed as mean ± SEM (n = 3, **p < 0.01, ***p < 0.001 vs unstimulated). [file mmc3.pdf]
